# Effects of SCN1A and SCN2A polymorphisms on responsiveness to valproic acid monotherapy in epileptic children: A protocol for systematic review and meta-analysis

**DOI:** 10.1097/MD.0000000000025831

**Published:** 2021-05-21

**Authors:** Zhuangfei Wen, Jiang Chen, Bin Zhu, Yan Lu, Lijiao Chen

**Affiliations:** a Department of Child Rehabilitation; b Department of Pediatrics, Haikou Hospital of the Maternal and Child Health, Haikou, Hainan Province, China.

**Keywords:** antiepileptic, children, epileptic, meta-analysis, protocol, SCN1A, SCN2A, valproic acid

## Abstract

**Background:**

: The gene mutation of coding sodium channel is one of the most important mechanisms in the pathogenesis of epilepsy. There exists a large inter-individual variation in the efficacy of valproic acid (VPA) against epilepsy. What are the genetic polymorphism influences of sodium channels on VPA response is still under discussion. In this study, a meta-analysis was used to further explore the effects of SCN1A and SCN2A gene polymorphism on VPA response in children with epilepsy.

**Methods:**

: The PubMed, EMBASE, Web of Science, Chinese National Knowledge Infrastructure, Chinese Science and Technique Journals Database, China Biology Medicine disc, and Wan Fang Database were searched up to April 2021 for appropriate studies regarding the association between SCN1A and SCN2A gene polymorphism on VPA response in children suffering from epilepsy. The meta-analysis was conducted by Review Manager 5.3 software.

**Results:**

: The results of this meta-analysis will be submitted to a peer-reviewed journal for publication.

**Conclusion:**

: This meta-analysis will summarize the effects of SCN1A and SCN2A gene polymorphisms on VPA response in children with epilepsy.

**OSF Registration Number:**

DOI 10.17605/OSF.IO/N2786.

## 1 Introduction

Epilepsy is a common disease in the nervous system of children.^[1–3]^ The incidence rate of epilepsy is 41/1 000 000 to 187/1 000 000.^[4]^ Antiepileptic drugs are commonly used for the treatment of epilepsy, but 30% of patients still relapse after obtaining adequate and adequate treatment.^[5]^ As a non-selective sodium channel blocker, sodium valproate can down-regulate the Scn3a gene expression of sodium channel through epigenetic pathway.^[6]^ Sodium valproate is a first-line drug for the treatment of epilepsy, and it has good effects on the control of generalized and local seizures, while these effects are greatly affected by genetic variation.^[7,8]^

Voltage-gated sodium channel is a common target of antiepileptic drugs.^[9,10]^ Mutations in SCN1A and SCN2A genes encoding the α subunit of ion channel are closely related to the response rate and drug resistance of valproic acid (VPA) and carbamazepine.^[11]^ Therefore, it is of great significance to improve the antiepileptic response rate of VPA from the point of genetics, the prognosis of patients and the rehabilitation of neurological functions.

At present, the relationship between SCN1A and SCN2A polymorphisms and the efficacy of VPA in the treatment of epilepsy in children are still controversial.^[12–15]^ So far, no meta-analysis on the relationship between SCN1A and SCN2A polymorphisms and the response of VPA to childhood epilepsy can be detected. Therefore, we performed a meta-analysis to clarify the relationship between SCN1A and SCN2A polymorphisms and the efficacy of VPA in the treatment of childhood epilepsy.

## 2 Methods

### 2.1 Study registration

The protocol of this review was registered in OSF (OSF registration number: DOI 10.17605/OSF.IO/N2786). According to some reports, it follows the statement guidelines of preferred reporting items for systematic reviews and meta-analyses protocol.^[16]^

### 2.2 Inclusion criteria

Literatures that meet the following criteria are included in this study:

1. patients, aged less than 18 years old, were diagnosed as epilepsy by EEG and imaging examination.
2. The study evaluated the effects of SCN1A and SCN2A polymorphism on the response to VPA treatment in children with epilepsy;
3. cohort study;
4. patients were treated with VPA;
5. data on the relationship between polymorphism and the efficacy of VPA can be obtained or deduced from original articles or corresponding authors.

### 2.3 Exclusion criteria

The exclusion criteria are as follows: case reports, meta-analysis, review articles, and studies without detailed genotype data.

### 2.4 Search strategy

Two researchers independently performed a systematic literature search in 7 electronic databases. The retrieval database includes the PubMed, EMBASE, Web of Science, Chinese National Knowledge Infrastructure, Chinese Science and Technique Journals Database, China Biology Medicine disc, and Wan Fang Database. The retrieval time was set to build the database until April 2021. The search strategy for PubMed is displayed in Table 1. This retrieval strategy will be used in other databases.

**Table 1:** Search strategy in PubMed database.

Number	Search terms
#1	Epilepsy [MeSH]
#2	Aura[Title/Abstract]
#3	Awakening Epilepsy[Title/Abstract]
#4	Epileptic Seizures[Title/Abstract]
#5	Seizure Disorder[Title/Abstract]
#6	Epilepsy, Cryptogenic[Title/Abstract]
#7	Seizures, Epileptic[Title/Abstract]
#8	Single Seizure[Title/Abstract]
#9	Auras[Title/Abstract]
#10	Cryptogenic Epilepsies[Title/Abstract]
#11	Cryptogenic Epilepsy[Title/Abstract]
#12	Epilepsies[Title/Abstract]
#13	Epilepsies, Cryptogenic[Title/Abstract]
#14	Epilepsy, Awakening[Title/Abstract]
#15	Epileptic Seizure[Title/Abstract]
#16	Seizure Disorders[Title/Abstract]
#17	Seizure, Epileptic[Title/Abstract]
#18	Seizure, Single[Title/Abstract]
#19	Seizures, Single[Title/Abstract]
#20	Single Seizures[Title/Abstract]
#21	or/1-20
#22	Child[MeSH]
#23	Children[Title/Abstract]
#24	or/22-23
#25	Valproic Acid[MeSH]
#26	Dipropyl Acetate[Title/Abstract]
#27	Divalproex[Title/Abstract]
#28	Sodium Valproate[Title/Abstract]
#29	2-Propylpentanoic Acid[Title/Abstract]
#30	Calcium Valproate[Title/Abstract]
#31	Convulsofin[Title/Abstract]
#32	Depakene[Title/Abstract]
#33	Depakine[Title/Abstract]
#34	Depakote[Title/Abstract]
#35	Divalproex Sodium[Title/Abstract]
#36	Ergenyl[Title/Abstract]
#37	Magnesium Valproate[Title/Abstract]
#38	Propylisopropylacetic Acid[Title/Abstract]
#39	Semisodium Valproate[Title/Abstract]
#40	Valproate[Title/Abstract]
#41	Valproate Sodium[Title/Abstract]
#42	Valproic Acid, Sodium Salt (2:1)[Title/Abstract]
#43	Vupral[Title/Abstract]
#44	2 Propylpentanoic Acid[Title/Abstract]
#45	Acetate, Dipropyl[Title/Abstract]
#46	Acid, Propylisopropylacetic[Title/Abstract]
#47	Acid, Valproic[Title/Abstract]
#48	Sodium, Divalproex[Title/Abstract]
#49	Sodium, Valproate[Title/Abstract]
#50	Valproate, Calcium[Title/Abstract]
#51	Valproate, Magnesium[Title/Abstract]
#52	Valproate, Semisodium[Title/Abstract]
#53	Valproate, Sodium[Title/Abstract]
#54	or/25-53
#55	NAV1.1 Voltage-Gated Sodium Channel[MeSH]
#56	Voltage-Gated Sodium Channel Type 1 Subunit alpha[Title/Abstract]
#57	NAV1.1 alpha Subunit[Title/Abstract]
#58	SCN1A Sodium Channel alpha Subunit[Title/Abstract]
#59	Sodium Channel, Voltage-Gated, Type I, alpha Protein[Title/Abstract]
#60	Type 1 Voltage-Gated Sodium Channel[Title/Abstract]
#61	Voltage-Gated Sodium Channel Type 1[Title/Abstract]
#62	Voltage-Gated Sodium Channel Type 1 alpha Subunit[Title/Abstract]
#63	NAV1.1 Voltage Gated Sodium Channel[Title/Abstract]
#64	Type 1 Voltage Gated Sodium Channel[Title/Abstract]
#65	Voltage Gated Sodium Channel Type 1[Title/Abstract]
#66	Voltage Gated Sodium Channel Type 1 Subunit alpha[Title/Abstract]
#67	Voltage Gated Sodium Channel Type 1 alpha Subunit[Title/Abstract]
#68	alpha Subunit, NAV1.1[Title/Abstract]
#69	SCN1A[Title/Abstract]
#70	NAV1.2 Voltage-Gated Sodium Channel[MeSH]
#71	Voltage-Gated Sodium Channel Type 2 Subunit alpha[Title/Abstract]
#72	NAV1.2 alpha Subunit[Title/Abstract]
#73	SCN2A Sodium Channel alpha Subunit[Title/Abstract]
#74	Sodium Channel Protein Type 2 Subunit alpha[Title/Abstract]
#75	Sodium Channel, Voltage-Gated, Type II, alpha 1[Title/Abstract]
#76	Sodium Channel, Voltage-Gated, Type II, alpha 1 Subunit[Title/Abstract]
#77	Type 2 Voltage-Gated Sodium Channel[Title/Abstract]
#78	Voltage-Gated Sodium Channel Type 2[Title/Abstract]
#79	Voltage-Gated Sodium Channel Type 2 alpha Subunit[Title/Abstract]
#80	NAV1.2 Voltage Gated Sodium Channel[Title/Abstract]
#81	Type 2 Voltage Gated Sodium Channel[Title/Abstract]
#82	Voltage Gated Sodium Channel Type 2[Title/Abstract]
#83	Voltage Gated Sodium Channel Type 2 Subunit alpha[Title/Abstract]
#84	Voltage Gated Sodium Channel Type 2 alpha Subunit[Title/Abstract]
#85	or/55–84
#86	Variation[Title/Abstract]
#87	Mutation[Title/Abstract]
#88	Polymorph∗[Title/Abstract]
#89	Variants[Title/Abstract]
#90	Variant[Title/Abstract]
#91	Susceptibility[Title/Abstract]
#92	or/86–91
#93	#21 and #24 and #54 and #85 and 92

### 2.5 Data collection and analysis

#### 2.5.1 Selection of studies

**Figure 1:**
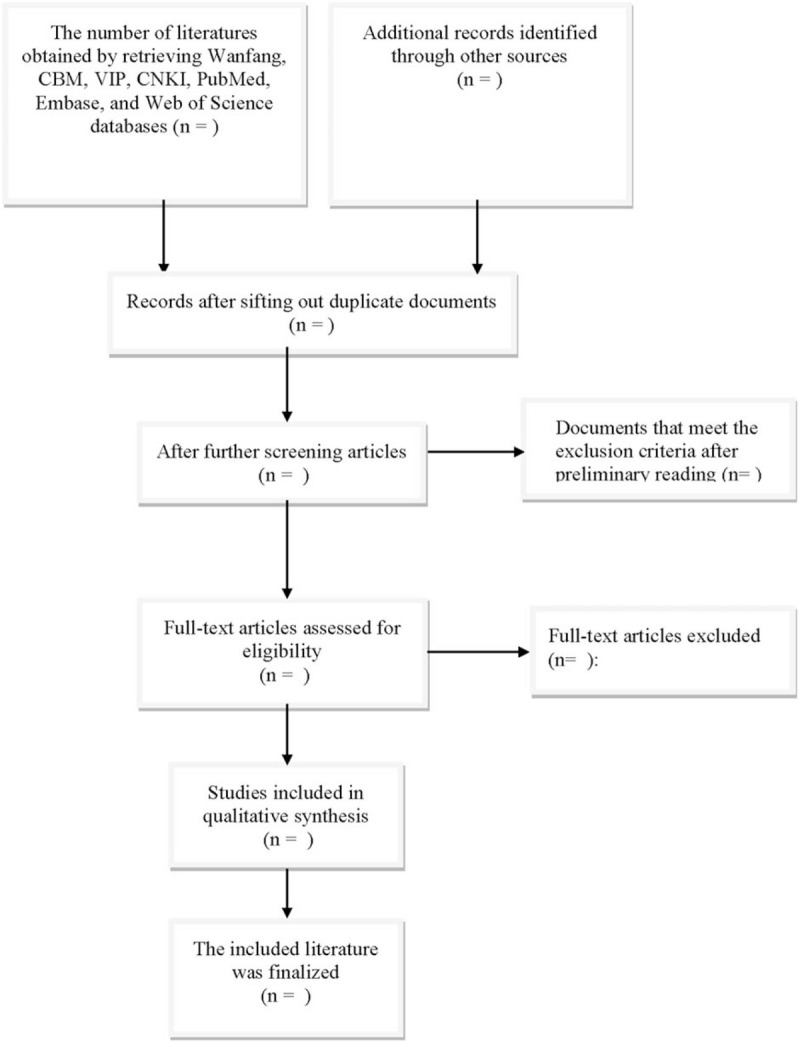
Flow diagram of study selection process.

The flowchart is demonstrated in Figure 1. The 2 researchers carried out literature screening and data extraction independently. Firstly, after excluding the obviously irrelevant literature, the title is read, and then the abstract and the full text are read to determine whether to include them or not. Any differences should be resolved by consulting a third party.

#### 2.5.2 Data extraction

In order to avoid bias in the process of data extraction, the 2 researchers adopted a blind method to extract data from the literature independently. The differences in the extracted data are figured out through negotiation and discussion. If necessary, some research data should be obtained directly from the corresponding authors. The following information was collected from each study: first author, year of publication, race, sample size, age, sex, efficacy evaluation criteria, genotypes, and the number of effective and ineffective people with different genotypes.

#### 2.5.3 Methodology quality assessment

We investigated the quality of each study based on the nine-point Newcastle-Ottawa Scale.^[17]^ The Newcastle-Ottawa scale values arrange from 0 to 9. Studies with the score of 6 are considered to be of high quality.^[18]^

#### 2.5.4 Dealing with missing data

If the data are not complete in the article, only descriptive analysis should be conducted, rather than meta-analysis.

#### 2.5.5 Statistical analysis

We calculated odds ratios and 95% confidence intervals for the analyses of the single-nucleotide polymorphisms and the efficacy of VPA. Five genotypic models, namely allele model (T vs C), heterozygote model (TC vs CC), homozygote model (TT vs CC), dominant model (TT + TC vs CC), and recessive model (TT vs TC + CC), were used to detect the relationship between single-nucleotide polymorphisms and response rate. The heterogeneity among studies was evaluated by performing Q-test and *I*^2^ statistics of Chi-Squared test. The significance level of heterogeneity threshold of Q test is 0.10, with *P* > .10. It can be considered that there is no heterogeneity among studies. *I*^2^ is used to measure the degree of heterogeneity among multiple research results. *I*^2^ values exhibit various degrees of heterogeneity in the range of 0% to 100% (0%–25%: no heterogeneity; 25%–50%: moderate heterogeneity; 50%–75%: large heterogeneity; 75%–100%: great heterogeneity). As long as *I*^2^ is less than 50%, its heterogeneity is acceptable. When *P* < .10 or *I*^2^ > 50%, the random effect model of the DerSimonian&Laird method is adopted to calculate the combined statistics. Otherwise, the fixed effect model of the Mantel-Haenszel method is applied for calculation. When the *P* value was bilateral, with *P* < .05, it was considered to be statistically significant. All analyses were carried out using Review Manager 5.3 software.

#### 2.5.6 Subgroup analysis

Through the review of the characteristics included in the study, the potential confounding factors were selected for subgroup analysis.

#### 2.5.7 Sensitivity analysis

The eligible study was sequentially removed to perform the sensitivity analysis.

#### 2.5.8 Assessment of publication biases

If no less than 10 studies are included, funnel charts are used to assess publication bias.^[19,20]^

#### 2.5.9 Ethics and dissemination

The content of this article does not involve moral approval or ethical review and would be presented in print or at relevant conferences.

## 3 Discussion

Voltage-gated sodium channel gene mutations may participate in the occurrence of epilepsy, thus affecting the response of individuals to antiepileptic drugs.^[21,22]^ Voltage-gated sodium channel consists of one α subunit and several β subunits, in which α subunit is the main structural and functional unit.^[23]^ Mutations in SCN1A and SCN2A genes of α subunit are associated with epilepsy in children.^[24,25]^ VPA may play an antiepileptic role by blocking sodium channels. Therefore, the mutation of sodium channel gene can lead to changes in the structure and function of the drug target, so that the drug cannot combine with the target, thus inhibiting the discharge of neurons and the emergence of drug resistance. Previous reports on the relationship between SCN1A and SCN2A gene mutations and the efficacy of VPA were inconsistent. In this study, meta-analysis was conducted to further explore the relationship between SCN1A and SCN2A gene polymorphisms and the efficacy of VPA in the treatment of childhood epilepsy, so as to provide etiological basis for individualized treatment of VPA in children with epilepsy.

## Author contributions

**Conceptualization:** Lijiao Chen.

**Data curation:** Zhuangfei Wen, Jiang Chen.

**Formal analysis:** Zhuangfei Wen.

**Funding acquisition:** Lijiao Chen.

**Methodology:** Bin Zhu.

**Project administration:** Lijiao Chen.

**Resources:** Bin Zhu.

**Supervision:** Lijiao Chen.

**Validation:** Bin Zhu, Yan Lu.

**Visualization:** Bin Zhu, Yan Lu.

**Writing – original draft:** Lijiao Chen, Zhuangfei Wen, Jiang Chen.

**Writing – review & editing:** Lijiao Chen, Zhuangfei Wen, Jiang Chen.
